# Berberine alleviates lipid metabolism disorders via inhibition of mitochondrial complex I in gut and liver

**DOI:** 10.7150/ijbs.54604

**Published:** 2021-04-12

**Authors:** Muyu Yu, Miriayi Alimujiang, Lili Hu, Fang Liu, Yuqian Bao, Jun Yin

**Affiliations:** 1 Department of Endocrinology and Metabolism, Shanghai Jiao Tong University Affiliated Sixth People's Hospital, Shanghai Clinical Center for Metabolic Diseases, Shanghai Key Laboratory of Diabetes Mellitus, Shanghai Diabetes Institute, 600 Yishan Road, Shanghai, 200233, China.; 2 Department of Endocrinology and Metabolism, Shanghai Eighth People's Hospital, Shanghai, 200235, China.

**Keywords:** dyslipidemias, NAFLD, diabetes, oxidative phosphorylation, lipid synthesis, fatty acid uptake

## Abstract

This study is to investigate the relationship between berberine (BBR) and mitochondrial complex I in lipid metabolism. BBR reversed high-fat diet-induced obesity, hepatic steatosis, hyperlipidemia and insulin resistance in mice. Fatty acid consumption, β-oxidation and lipogenesis were attenuated in liver after BBR treatment which may be through reduction in SCD1, FABP1, CD36 and CPT1A. BBR promoted fecal lipid excretion, which may result from the reduction in intestinal CD36 and SCD1. Moreover, BBR inhibited mitochondrial complex I-dependent oxygen consumption and ATP synthesis of liver and gut, but no impact on activities of complex II, III and IV. BBR ameliorated mitochondrial swelling, facilitated mitochondrial fusion, and reduced mtDNA and citrate synthase activity. BBR decreased the abundance and diversity of gut microbiome. However, no change in metabolism of recipient mice was observed after fecal microbiota transplantation from BBR treated mice. In primary hepatocytes, BBR and AMPK activator A769662 normalized oleic acid-induced lipid deposition. Although both the agents activated AMPK, BBR decreased oxygen consumption whereas A769662 increased it. Collectively, these findings indicated that BBR repressed complex I in gut and liver and consequently inhibited lipid metabolism which led to alleviation of obesity and fatty liver. This process was independent of intestinal bacteria.

## Introduction

Obesity is one of the most prevalent public health issues worldwide due to sedentary lifestyle and high-calorie diet, leading to hepatic steatosis, cardiovascular disease and diabetes with increasing morbidities 1, 2. Nevertheless, current drugs have limited effect on obesity and its related metabolic dysregulation 3-5. Berberine (BBR), an isoquinoline alkaloid, has been reported to improve glucose metabolism 6-8 as well as enhance insulin sensitivity 9, 10 in previous studies including ours. In addition, decreased body weight, cholesterol and triglyceride levels were also observed with BBR treatment 11, 12, which makes it a potential candidate for the treatment of obesity and lipid metabolism disorders. However, the underlying mechanism still remains ambiguous.

Mitochondrion is a cell organelle responsible for aerobic metabolism such as tricarboxylic acid cycle and oxidative phosphorylation (OXPHOS). Our previous research and some other studies have revealed that liver mitochondrial function enhanced in obese and diabetic mice 13-15, while BBR was able to reduce oxygen consumption of hepatocytes *in vitro* as well as enhance glucose consumption via inducing glycolysis 16-18. And evidences including ours also confirmed that BBR inhibited complex I function 17,19-21. Meanwhile, we have found that the mitochondrial complex I inhibitor rotenone (ROT) improved glucose homeostasis, which was similar as BBR 22. Nevertheless, previous studies mainly focused on the beneficial effect of complex I inhibition by BBR on glucose metabolism, the relationship between mitochondrial complex I function and lipid metabolism is yet poorly understood.

By β-oxidation, an aerobic biological process, fatty acids are broken into acetyl-CoA in mitochondria 23. Our previous study demonstrated that ROT inhibited OXPHOS of complex I and reduced the ratio of NAD^+^/NADH 22. NADH is a product of β-oxidation, therefore, we speculated that ROT and BBR may slow the rate of β-oxidation. However, several groups reported that BBR enhanced fatty acid oxidation by activating adenosine 5'-monophosphate-activated protein kinase (AMPK) pathway 24, 25. Thus, whether BBR increases or decreases fatty acid oxidation needs further clarification. If BBR inhibits it, how BBR suppresses lipid deposition is an interesting issue to explore.

It's known that intestinal microbiota has a profound impact on lipid metabolism as well 26, and BBR has been reported to improve dyslipidemia by modulating structure and diversity of the microbiota of high-fat-diet (HFD) mice 27, 28. Whereas without complete respiratory enzyme system, most intestinal bacteria are obligate anaerobes, may not respond to the inhibition of complex I. Whether the lipid-lowing effect of BBR attributing to mitochondrial complex I inhibition or intestinal microbiota alteration remains unclear. In addition to gut bacteria, some literatures showed that the gut itself played an important role in metabolism 29, 30. Nevertheless, up to now, little attention has been paid to the action of BBR on intestinal mitochondria.

To address above issues, we used ROT as a positive control to investigate the effects and mechanism of BBR on lipid metabolism disorders. The study indicated that BBR and ROT reduced body weight and hepatic lipid deposition of obese mice through decreasing mitochondrial oxygen consumption rate (OCR), ATP synthesis and intestinal lipid absorption. Additionally, the metabolic improvement was independent of alteration of gut microbiome by BBR.

## Materials and Methods

### Animals

All male C57BL/6J (Nanjing Biomedical Research Institute of Nanjing University, N000013) at 5 weeks of age were housed with constant temperature (22 ± 2 °C) and humidity (40-60%) as well as 12 h light/dark cycle. The mice had free access to diet and water except for indicated fasting conditions. Forty mice were randomly divided into four groups and fed with normal chow diet (NCD), HFD, HFD+BBR and HFD+ROT (n = 10), respectively. NCD and HFD were both purchased from Trophic Animal Feed High-tech Co., Ltd. (China), in which 10% and 60.9% of calories were from fat, respectively. BBR (A600129, Sangon) and ROT (R8875, Sigma) were blended into the HFD at 1.4 g/kg and 0.075 g/kg, respectively. The diet was stored in a -20 °C freezer until use. The cages and food were changed every three days. RER and heat were accessed by Oxymax indirect calorimetry system (Oxymax, Columbus Instruments). Dual energy X-ray absorptiometry (GE PIXImus1) was used to measure body composition. After 5 months of feeding, animals were sacrificed with 1% sodium pentobarbital anesthesia (10 µl/g) and tissues were collected with liquid nitrogen and stored at -80°C until use. Meanwhile, liver tissues were put in 4% paraformaldehyde to perform HE and oil red staining.

### Intraperitoneal glucose tolerance test (IPGTT) and insulin tolerance test (ITT)

IPGTT was performed with intraperitoneal injection of glucose (2 g/kg) after overnight fasting. Blood glucose was determined at 0, 15, 30, 60 and 120 min after glucose injection. ITT was carried out by intraperitoneal injection of regular insulin (0.75 unit/kg, Novo Nordisk) after 6 h fasting. Blood glucose was measured at 0, 15, 30, 60 and 120 min after insulin injection.

### Oxygen consumption of tissue mitochondria

Intact liver and gut mitochondria were isolated as previously described 31. Mitochondrial protein concentration was measured by Bradford assay kit (Beyotime). A clark-type oxygen electrode (Strathkelvin Instruments, Motherwell, UK) was used to assess mitochondrial OCR. The tests were carried out in oxygen electrode gimish (50 mmol/L Mops, 100 mmol/L KCl, 5.0 mmol/L KH_2_PO4, 1.0 mmol/L EGTA, 1 mg/ml defatted BSA, pH 7.4). Glutamate (20 mmol/L) + malate (5 mmol/L), ROT (7.5 μmol/L) + succinate (20 mmol/L) and ROT (7.5 μmol/L) + TMPD-Asc (1 mmol/L -10 mmol/L) were added as respiratory substrates for mitochondrial complex Ⅰ, Ⅱ and Ⅳ, respectively. After mitochondria and 0.1 mmol/L ADP were added, State 3 respiration started when related substrates and 0.2 mmol/L ADP were injected. Subsequently, we assessed OXPHOS capacity and electron transport chain (ETC) capacity with adding 2 mmol/L ADP and 0.2 mmol/L DNP (uncoupler), respectively. The data of OCRs were shown as nM atoms of O_2_/minute/mg of mitochondrial protein.

### Carnitine acyl transferase activity

Carnitine acyl transferase activity was assessed by OCR using a clark-type oxygen electrode, which was prepared as mentioned above. We used carnitine + palmitoyl CoA to assess carnitine acyl transferase (CPT) 1+2 activity and palmitoyl carnitine to measure CPT2 activity alone. After mitochondria and 0.1 mmol/L ADP were injected, state 3 respiration was initiated when adding substrates and 0.2 mmol/L ADP. OXPHOS capacity was measured by 2 mmol/L ADP, mimicking the maximum extent of fatty acyl CoA being transported.

### Complex III activity

The activity of cytochrome c reductase (complex III) was measured by the reduction of cytochrome c as previously described with a little modification 32. Briefly, the enzyme assay was carried out in buffer complemented with 50 mmol/L KPi, 50 μmol/L EDTA, 0.1% BSA, 10 μg/mL antimycin A, 0.1 mmol/L decylubichinone and 60 μmol/L cytochrome c. The reaction was started with the administration of 50 mg liver or intestine tissues and the reduction of cytochrome c was evaluated at a wavelength of 550 nm.

### Citrate synthase and β-ketothiolase activities

Activities of citrate synthase (CS) and β-ketothiolase were assessed as previously described with a little modification 33. Mitochondria were pretreated with 5% cholate (pH 7) before adding to the reagent cocktail.

### Transmission electron microscopy (TEM)

Liver tissues were collected after mice were sacrificed immediately and divided into sections of 1 mm × 1 mm × 1 mm approximately, which were fixed with glutaraldehyde and osmic acid, dehydrated with ethanol and embedded with ethoxyline resin. And then we observed mitochondrial ultrastructural morphology and structure by TEM. Mitochondrial density, length (the most extended dimension of mitochondria) and area were analyzed in 5 microscopic vision fields (13500×) by Image J.

### Isolation and treatment of mouse primary hepatocytes

Primary hepatocytes were isolated from male C57BL/6J mice of 6-8 weeks by a previously described two-step perfusion method with minor modifications 34-36. We seeded primary hepatocytes on geltin-coated 6- or 12-well plates in DMEM (low glucose) supplemented with 10% fetus bovine serum and 1% penicillin-streptomycin (Gibco, Grand Island, NY, USA). Trypan blue was used to make sure that cell viability was higher than 80% before plating. Primary hepatocytes were cultured in a humidified atmosphere of 95% air and 5% CO2 at 37℃. After 4-6 h adherence, cells were treated with 0.8 mmol/L oleic acid (OA) to induce lipid accumulation, supplemented with 1, 2, 4 μmol/L BBR or 0.5, 1, 2 μmol/L ROT in serum-free DMEM for 24 h. When exploring the role of AMPK pathway in the effect of BBR, we incubated primary hepatocytes with 10 μmol/L compound C (CC) or 20 μmol/L A769662 (A76) to block or activate AMPK activity. Lipid droplets of primary hepatocytes were monitored by oil red O staining. We also measured intracellular triglyceride using Triglyceride Quantification Colorimetric/Fluorometric Kit (BioVision). ATP contents of primary hepatocytes were assessed by Luminescent ATP Detection Assay Kit (Abcam).

### LDH cytotoxicity assay

After OA, BBR and ROT treatment for 24 h, supernatant of primary hepatocytes was collected to measure cytotoxicity with LDH Cytotoxicity Assay Kit (Beyotime), which was calculated by the following equation: living cells (%) = 100 - (OD490_treated_ - OD490_blank_) / (OD490_maximum enzyme activity_- OD490_blank_) × 100%.

### *In vitro* lipid assays

After primary hepatocytes were treated with OA, OA+BBR and OA+ROT for 24 h, we collected the supernatant of each group and assessed the non-esterified free fatty acids (Wako). And the results were normalized to the NEFA amounts of OA-treated blank well (cell free) to evaluate the fatty acid uptake with a colorimetric assay.

For cellular triglyceride assay, we incubated primary hepatocytes with different treatment for 24 h, followed by lysing the cells in 5 % NP-40. The samples were heated to 95 °C for 5 min and cooled down to room temperature. After being centrifuged, the collected supernatant was used to determine the triglyceride level (BioVision).

### Oxygen consumption of primary hepatocytes

Primary hepatocytes were seeded into XFe-24 cell plates. After the cells were incubated with different treatment for 24 h, we removed the medium and washed the cells twice. Then Seahorse assay medium was added to plate and incubated for 1 h at 37 °C without CO_2_. Next, oligomycin, FCCP, antimycin and ROT were pretreated into reagent delivery ports of A, B, and C, respectively. Then OCR were measured by Seahorse XFe-24 analyser (Agilent technologies). The basal respiration was calculated by the baseline of OCR and maximal respiration was assessed by OCR after FCCP injection.

### *In vivo* biochemical analyses

After being fed with NCD, HFD, HFD+BBR and HFD+ROT for 24 weeks, the mice were food deprived for 12 h, and then they received euthanasia to collect plasma by cardiac puncture. Plasma triglyceride (BioVision), cholesterol (Wako), alanine aminotransferase (ALT, Thermo Scientific), creatinine, urea, HDL-C and LDL-C (Kehua) were measured by colorimetric assay kits according to the manufacturer's instructions.

The fecal lipid analysis was carried out as described previously 37 by ICAS Testing Center (Shanghai, China). In brief, dried fecal samples of each groups were collected and lipids were extracted by 2:1 chloroform-ethanol. The dried lipid extracts were resuspended in 1% Triton X-100 dissolved in chloroform and evaporated overnight. After finally suspended in water, the lipid contents were measured.

### mtDNA assay

DNA of the liver tissues was extracted with DNeasy Blood & Tissue Kit (QIGEN, Hilden, Germany) according to the manufacturer's instructions. Total DNA (25 ng) was used as a template in RT-PCR. The mtDNA primers used for PCR were listed as follows: MT-ND1 (Forward, CTAACAACTATTATCTTCCTAGGAC; Reverse, GATGTATAAGTTGATCGTAACGG); MT-RNR1 (Forward, AGGAGCCTGTTCTATAATCGATAAA; Reverse, GATGGCGGTATATAGGCTGAA). All above mtDNA levels were normalized to the nuclear RBM15 (RNA-binding motif protein 15) gene (Forward, GGACACTTTTCTTGGGCAAC; Reverse, AGTTTGGCCCTGTGAGACAT).

### 16S sequencing

Total DNA was extracted from the feces by QIAamp Fast DNA Stool Mini Kit (Qiagen). The microbial 16S rRNA gene V5-V6 region of the samples was amplified with forward primer 786F (5′- GATTAGATACCCTGGTAGT-3′) and the reverse primer 1079R (5′- TCACGACACGAGCTGACGAC-3′) by PCR and Illumina TruSeq DNA PCR-Free Sample Preparation Kit (Illumina) was used to construct the DNA library. Sequencing was carried out by an Illumina MiSeq platform. To check on the quality of raw data, Fast-QC (http://www.bioinformatics.babraham.ac.uk/projects/fastqc) was utilized for quality control. Quantitative Insights into Microbial Ecology (QIIME v.1.9.3, https://qiime.org/) was used to identify and remove chimeric sequences.

Sequences were grouped into operational taxonomic units (OTUs) at 97% similarity and aligned against the Greengenes reference database. The Chao1 index and Shannon index were carried out to evaluated the species richness and diversity.

### Fecal microbiota transplantation (FMT)

Six-week-old male donor mice were fed with HFD, HFD+BBR, or HFD+ROT (n = 5) for 4 months. After 8 weeks of feeding, stools were collected daily for the subsequent 2 months under a laminar flow hood in sterile condition. Stools (100-150 mg) from donor mice of each diet group were pooled and resuspended in 3 ml of sterile saline. The solution was vigorously mixed for 30 s. After standing for 5 minutes, 300 µl supernatant of the solution was collected and orally administered into each recipient. All recipients were fed with HFD. FMT was repeated twice a week for consecutive 8 weeks. Meanwhile, feces excreted by donor mice were also dropped to the recipients' cages because mice were coprophilous.

### Western blot

Primary hepatocytes, liver and intestine tissues were collected and lysed in RIPA buffer containing PMSF and phosphatase inhibitor cocktail for 10 min on ice. After determining protein concentrations, we boiled the lysates and subjected them to SDS-PAGE. Subsequently, proteins were transferred onto polyvinylidene fluoride membranes, which were blocked with 5% defatted milk for 1 h at room temperature. The membrane was incubated overnight at 4 °C with primary antibodies and then incubated with secondary antibodies for 1 h at room temperature. Immunoreactive signals were detected with ECL reagent (Thermo Scientific). Image J was used to quantify the Western bands.

Antibodies against SCD1 (2794), FABP1 (13368), CS (14309), COX IV (11967), AMPKα (5832), p-AMPKα (Thr172, 2535), ACC (3662), p-ACC (Ser79, 3661), GAPDH (5174), β-actin (3700), HSP90 (4877), anti-mouse (7076) and anti-rabbit (7074) secondary antibodies were obtained from Cell Signaling Technology. Antibodies to CPT1A (ab128568), ACADL (ab196655), FADS1 (ab126706), mt-ND3 (ab192306), CD36 (ab133625) and GRIM19 (ab110240) were purchased from Abcam. Antibody against Ndufs4 (WH0004724M1) was from Sigma. Antibody to mt-ND2 (LS-C498022) was acquired from Lifespan.

### Statistical analysis

Data were shown as means ± SEM. Statistical significance of differences was determined by one-way or two-way ANOVA with Tukey-Kramer multiple comparisons tests. A* P* value of less than 0.05 was considered to be statistically significant and the statistical analysis was carried out with SPSS 20.0.

## Results

### BBR prevented obesity and insulin resistance in HFD mice

**Figure 1:**
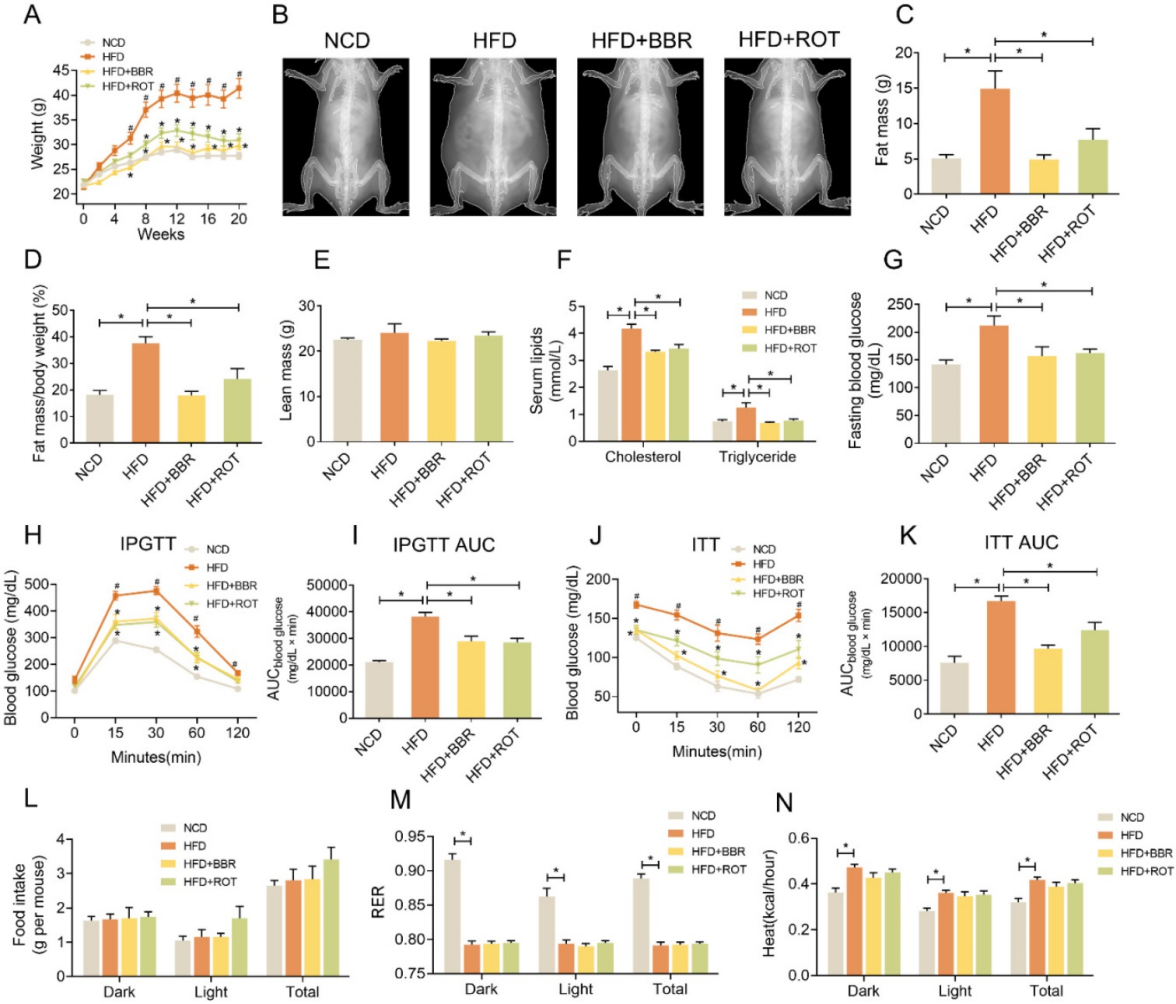
** BBR reduced body weight and improved glucose and lipid metabolism of HFD mice.** (A) Body weight of the mice fed with NCD, HFD, HFD+BBR and HFD+ROT for 20 weeks (*n* = 10). (B-F) The gross morphology (B), fat mass (C), fat mass/body weight (D) and lean body mass (E) of the mice by a dual-energy X-ray absorptiometry (*n* = 4). (F-K) Blood lipids (F), fasting blood glucose (G), IPGTT curve (H) and AUC (I), ITT curve (J) and AUC (K) (F、G:* n* = 6; H-K: *n* = 10). (L-N) Food intake (L), RER (M) and heat (N) assessed by metabolic cages (*n* = 8). Data were expressed as means ± SEM. *P < 0.05.

Mice were fed with NCD, HFD, HFD+BBR and HFD+ROT, respectively. From the 6th week to the 20th week, the body weights of the HFD group were much higher than those of the other groups (Figure 1A and B), while the fat mass and fat mass/body weight of the HFD+BBR and HFD+ROT groups were in low levels similar as NCD group (Figure 1C and D). No significant difference in lean mass was observed among the groups (Figure 1E). BBR and ROT significantly decreased the serum cholesterol, triglycerides, fasting blood glucose (FBG) and alanine aminotransferase (ALT) which increased markedly with HFD (Figure 1F and G, Table S1).

Notably, IPGTT showed that HFD impaired glucose tolerance of the mice while BBR and ROT reversed these changes (Figure 1H and I). BBR also significantly decreased glucose levels and AUC in ITT (Figure 1J and K). Food intake of the four groups was similar (Figure 1L), indicating that BBR and ROT reduced body weight of the obese mice without affecting calorie intake. In addition, HFD decreased respiratory exchange rate (RER) and increased the heat of mice compared with NCD, and no influence of BBR or ROT was observed on the effects of HFD (Figure 1M and N).

### BBR reduced lipid deposition in hepatocytes

**Figure 2:**
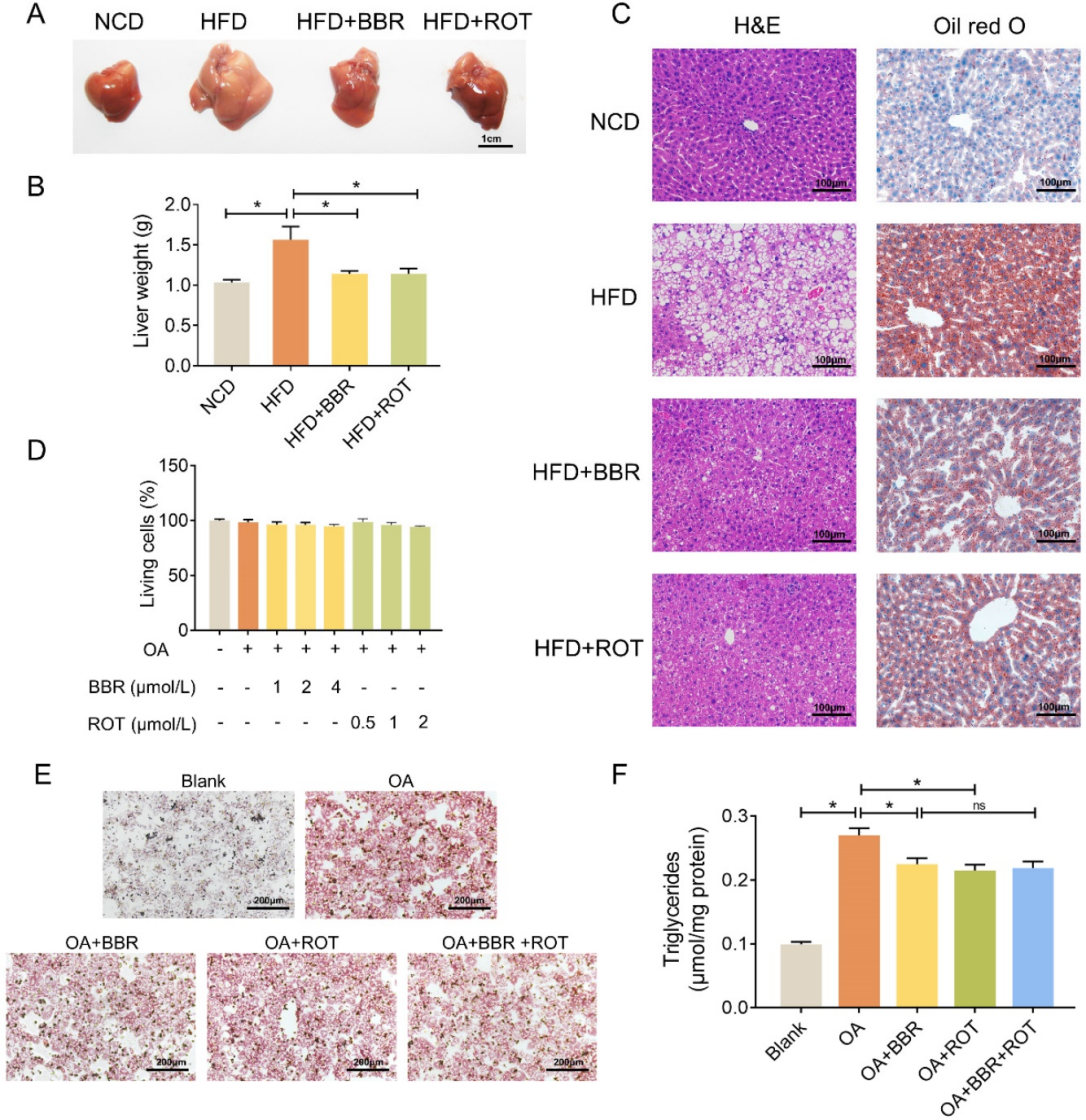
** BBR alleviated lipid deposition in hepatocytes both *in vivo* and *in vitro*.** (A-C) Lipid deposition of the mice was assessed by gross morphology (A), liver weight (B), HE staining and oil red staining (C) of liver tissue (*n* = 10). (D) Drug concentration screening of primary hepatocytes by cytotoxicity. (E-F) Oil red staining (E) and intracellular triglyceride content (F) of the primary hepatocytes incubated with blank, OA, OA+BBR, OA+ROT or OA+BBR+ROT for 24 h (*n* = 4). Data were expressed as means ± SEM. *P < 0.05.

As shown in Figure 2A and B, fatty liver induced by HFD was restored by BBR and ROT. Concurrently, HE and oil red O staining showed that BBR and ROT markedly reduced the lipid deposition in the liver of obese mice (Figure 2C). *In vitro*, with 0.8 mmol/L OA to induce the steatosis of primary hepatocytes, LDH release test was utilized to determine the drug concentration and no cytotoxicity was observed in BBR (1 μmol/L, 2 μmol/L and 4 μmol/L) or ROT (0.5 μmol/L, 1 μmol/L and 2 μmol/L) treated mouse primary hepatocytes (Figure 2D). Therefore, we used 4 μmol/L BBR and 2 μmol/L ROT for the following experiments. Oil red staining revealed a reduction in lipid droplets of hepatocytes in HFD+BBR and HFD+ROT groups. In accordance, BBR or ROT administration for 24 h significantly decreased triglyceride content in OA treated hepatocytes. Whereas, no differences were observed between OA+BBR and OA+BBR+ROT groups, which indicated that the addition of ROT could not exert more pronounced lipid-lowering effect on the basis of BBR treatment (Figure 2E and F). Together, these data proved that BBR and ROT relieved lipid accumulation in hepatocyte steatosis.

### BBR suppressed fatty acid uptake, lipid synthesis and fatty acid oxidation in liver

**Figure 3:**
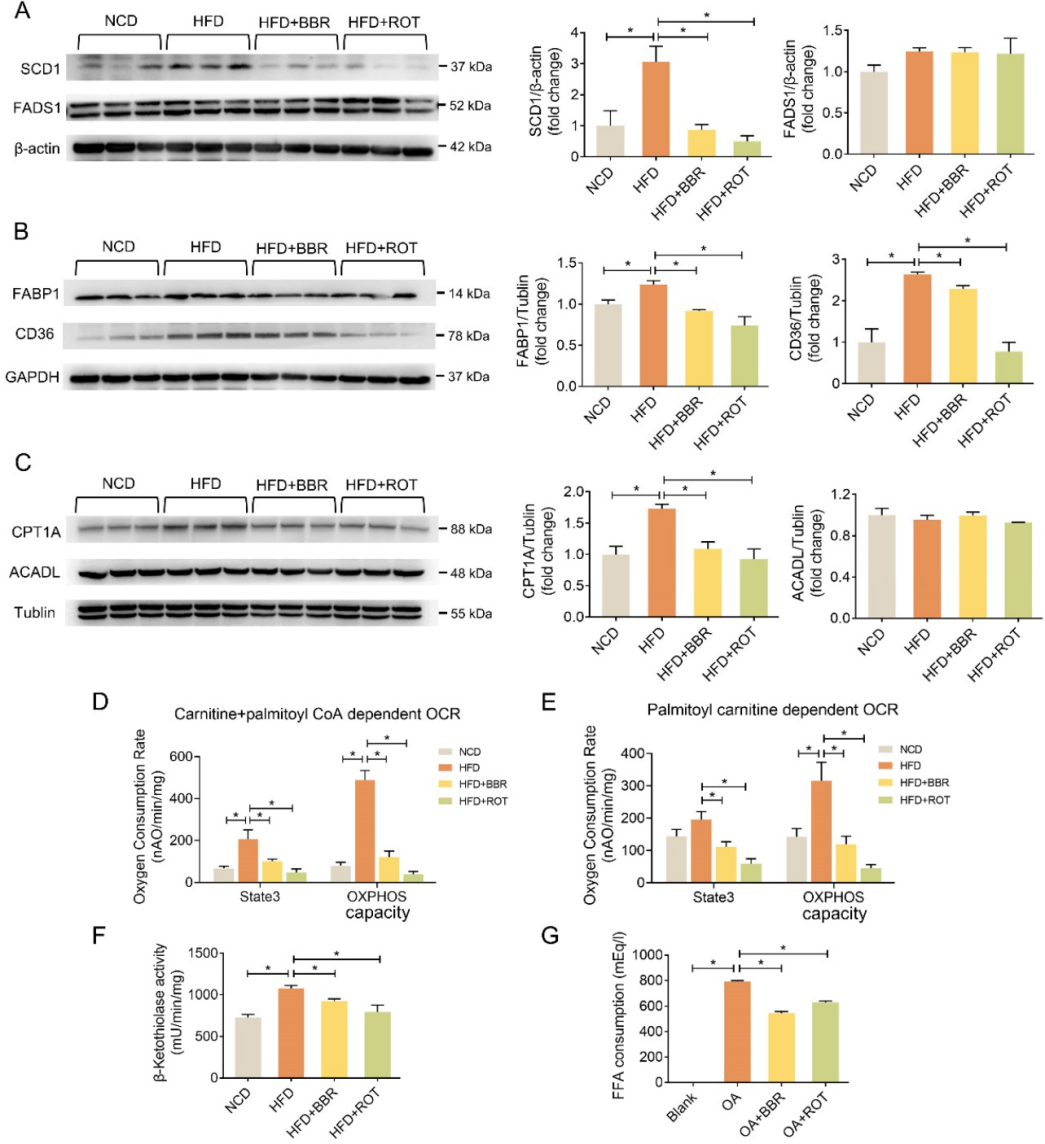
** BBR inhibited lipogenesis, fatty acid uptake and fatty acid oxidation of liver tissues.** (A-C) The protein levels of representative key-enzymes in lipid pathways of liver tissues, such as SCD1 and FADS1 for lipogenesis (A), FABP1 and CD36 for fatty acid uptake (B), as well as CPT1A and ACADL for fatty acid oxidation (C). Relative signal strength was quantified for each band (*n* = 3). (D-F) Mouse liver mitochondria of carnitine + palmitoyl CoA dependent OCR indicating CPT1+2 activities (D), palmitoyl carnitine dependent OCR indicating CPT2 activity (E), and β-ketothiolase activity indicating β-oxidation activity (F) (*n* = 6). (G) Fatty acid consumption in the primary hepatocytes after the cells were incubated with blank, OA, OA+BBR and OA+ROT for 24 h (*n* = 4). Data were expressed as means ± SEM. *P < 0.05.

The effects of BBR on the protein levels of lipid metabolic pathways were examined by Western blot. As shown in Figure 3A-C, HFD significantly induced the expression of key enzymes in hepatic lipid synthesis (SCD1), fatty acid uptake (FABP1 and CD36) and fatty acid oxidation (CPT1A) whereas BBR and ROT reversed these over-expressed protein levels. Meanwhile, no significant difference among the four groups was observed in the other key enzymes of lipid metabolism, e.g. FADS1 and ACADL, indicating that BBR and ROT did not alter the protein levels which were not affected by HFD.

OCR of fatty acid oxidation in liver mitochondria with carnitine and fatty acyl-CoA as substrates was measured to reflect the activity of CPT1+2, key enzymes of fatty acyl-CoA transport. CPT2 activity alone was tested with acyl-carnitine used as a substrate for OCR. BBR and ROT significantly inhibited the activities of CPT1 and 2 (Figure 3D and E). Furthermore, BBR and ROT decreased the activity of β-ketoacyl-CoA thiolase, a subunit of mitochondrial trifunctional protein in charge of β-oxidation of long-chain fatty acids (Figure 3F).

To investigate the activity of BBR in fatty acid uptake, we examined the reduction of OA in the culture medium of primary hepatocytes treated with OA+BBR or ROT. The results indicated that the two drugs significantly suppressed fatty acid consumption (Figure 3G).

### BBR inhibited liver mitochondrial complex I-dependent OCR and ATP synthesis

**Figure 4:**
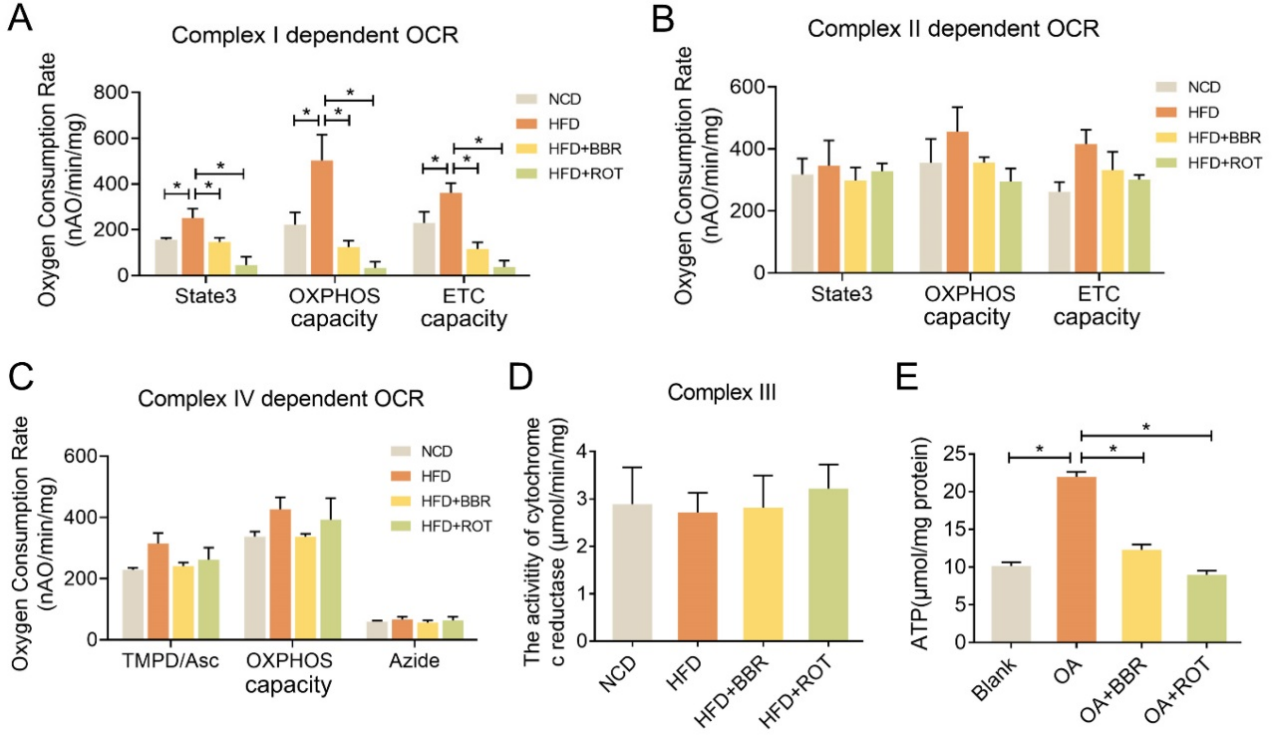
** BBR inhibited mitochondrial complex I dependent OCR and ATP synthesis.** (A-C) Complex I dependent OCR (A), complex II dependent OCR (B), complex IV dependent OCR (C) of mouse liver mitochondria (*n* = 3). (D) The activity of cytochrome c reductase (complex III) of liver tissues (*n* = 3). (E) ATP content of primary hepatocytes incubated with blank, OA, OA+BBR and OA+ROT for 24 h (*n* = 8). Data were expressed as means ± SEM. *P < 0.05.

To evaluate the effect of BBR on mitochondrial function, OXPHOS of the liver mitochondria extracted from the four groups was determined in the presence of different complex substrates. HFD notably stimulated the OCRs of state3, OXPHOS capacity and ETC capacity in complex I, showing no significant impacts on complex II and IV activities. BBR completely reversed the overactivation of complex I function of HFD group and had minimal effect on complex II and IV. Specifically, compared with HFD group, oral administration of BBR decreased complex I dependent OCR of state3, OXPHOS capacity and ETC capacity by 56.5%, 81.3% and 75.8%, respectively (Figure 4A-C). Additionally, the activity of cytochrome c reductase was carried out to assess complex III activity and there were no apparent differences among the groups (Figure 4D). Moreover, ATP content in the primary hepatocytes of the OA+BBR and OA+ROT groups decreased by 43.6% and 55.8% compared with OA group, respectively (Figure 4E). These data indicated that the inhibition of complex I activity resulted in reduced intracellular ATP content.

### BBR stimulated liver mitochondrial fusion

**Figure 5:**
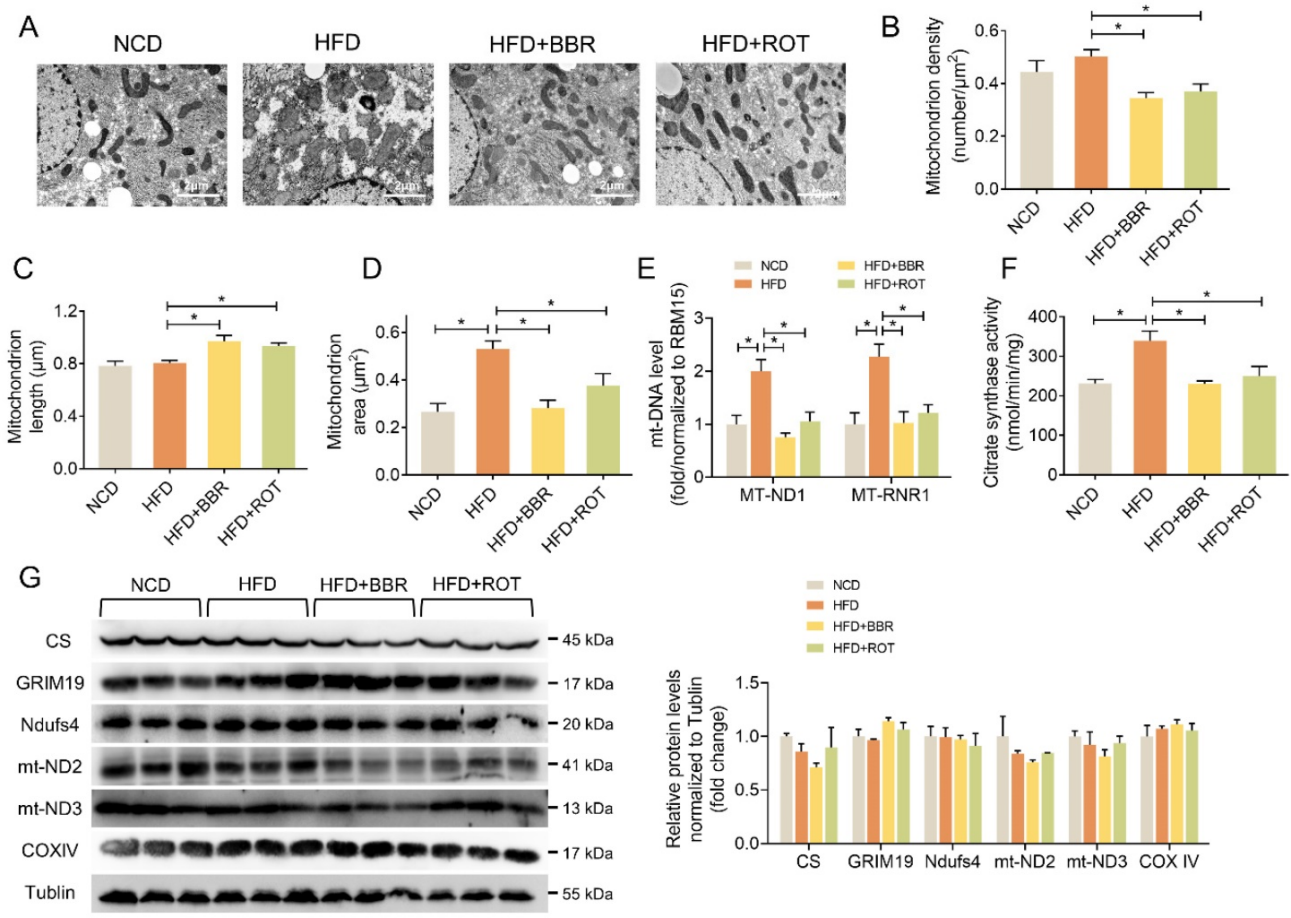
** BBR and ROT promoted liver mitochondrial fusion.** (A) Morphology and structure of mitochondria in liver with TEM. (B-D) The mitochondrial density (B), length (C) and area (D) by the statistical analyze of TEM (*n* =10). (E) Hepatic mtDNA copies (*n* = 7). (F) Citrate synthase activity of liver mitochondria (*n* = 6). (G) The protein levels of electron transport chain genes (CS, GRIM19, Ndufs4, mt-ND2, mt-ND3 and COXIV) by Western blot and relative signal strength quantification (*n* =3). Data were expressed as means ± SEM. *P < 0.05.

The influence of BBR on liver mitochondrial morphology and structure was explored by TEM. The images showed that BBR and ROT increased the mitochondrial length and decreased the density (Figure 5A-C). Meanwhile, HFD induced mitochondrial swelling as evidenced by doubled mitochondrial area without alteration of mitochondrial length. BBR and ROT completely reversed the mitochondrial swelling by HFD (Figure 5D). In addition, HFD remarkably increased the copy number of mtDNA, e.g. MT-ND1 and MT-RNR1, while BBR and ROT made them return to normal levels (Figure 5E). Citrate synthase is a pace-making enzyme in the first step of the citric acid cycle. As illustrated in Figure 5F, HFD significantly stimulated the activity of citrate synthase, which was back to normal level after BBR and ROT treatment. The above results suggested that BBR reduced the number of liver mitochondria and promoted their fusion. Moreover, no significant difference among the groups was observed on the protein levels of electron transport chain such as GRIM19, Ndufs4, mt-ND2, mt-ND3 and COXIV (Figure 5G).

### BBR activated AMPK pathway and alleviated lipid deposition

**Figure 6:**
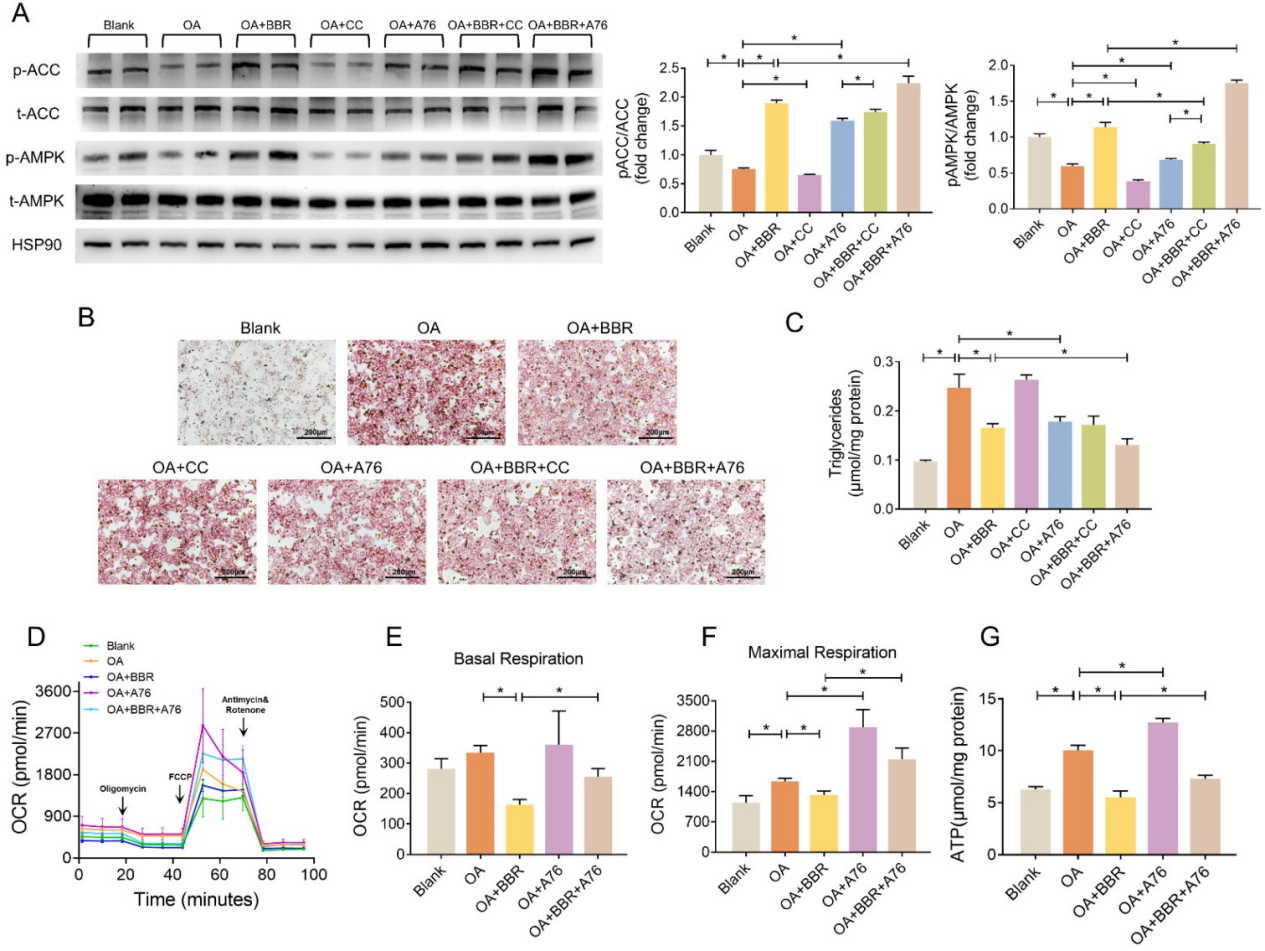
** BBR activated AMPK pathway.** (A) Phosphorylation of AMPK and ACC in the primary hepatocytes after blank, OA, OA+BBR, OA+CC, OA+A76, OA+BBR+CC or OA+BBR+A76 treatment by Western blot and relative signal strength quantification (*n* = 3). (B-C) Intracellular triglyceride content (B) and oil red staining (C) of the hepatocytes incubated with different treatment (*n* = 5). (D) OCR determined by Seahorse XFe-24 in primary hepatocytes responding to oligomycin, FCCP, antimycin, and rotenone (*n* = 3). (E-F) OCR of basal and maximal respiration calculated from the real-time oxygen consumption curve presented in (D). (G) ATP content of primary hepatocytes treated with blank, OA, OA+BBR, OA+A76 and OA+BBR+A76 (*n* = 8). Data were expressed as means ± SEM. *P < 0.05.

We further investigated whether AMPK played a role in the regulation of BBR on lipid metabolism disorders by A769662 (A76) and compound C (CC), an agonist and an inhibitor of AMPK, respectively. In the primary hepatocytes, OA significantly inhibited phosphorylation of AMPK and ACC, while BBR and A76 induced it markedly and BBR+A76 group had the highest phosphorylation levels of AMPK and ACC. CC decreased p-AMPK and p-ACC, which were increased by BBR+CC (Figure 6A). Additionally, BBR and A76 alleviated the lipid droplets and triglyceride content of the hepatocytes increased by OA, which were barely affected by CC. The concomitant application of BBR and A76 led to less lipid droplets and triglyceride content compared with the separate applications of the two drugs, possibly resulting from the synergic effect (Figure 6B and C). These data suggested that AMPK activity might be associated with the hypolipidemic effects of BBR.

Next, we performed Seahorse to evaluate the mitochondrial OCR of primary hepatocytes. Compared with OA group, BBR treatment decreased basal and maximal respiration by 51.1% and 19.4%, respectively. A76 increased the maximal respiratory capacity with no impact on basal respiration. The OCRs of basal and maximal respiration in OA+BBR+A76 group were between the ones in OA+BBR and OA+A76 group (Figure 6D-F). What's more, increased ATP content was observed in primary hepatocytes incubated with OA+A76, which was reduced by OA+BBR+A76 administration (Figure 6G).

### BBR reduced fat absorption via inhibiting intestinal mitochondrial complex I activity regardless of gut bacteria

**Figure 7:**
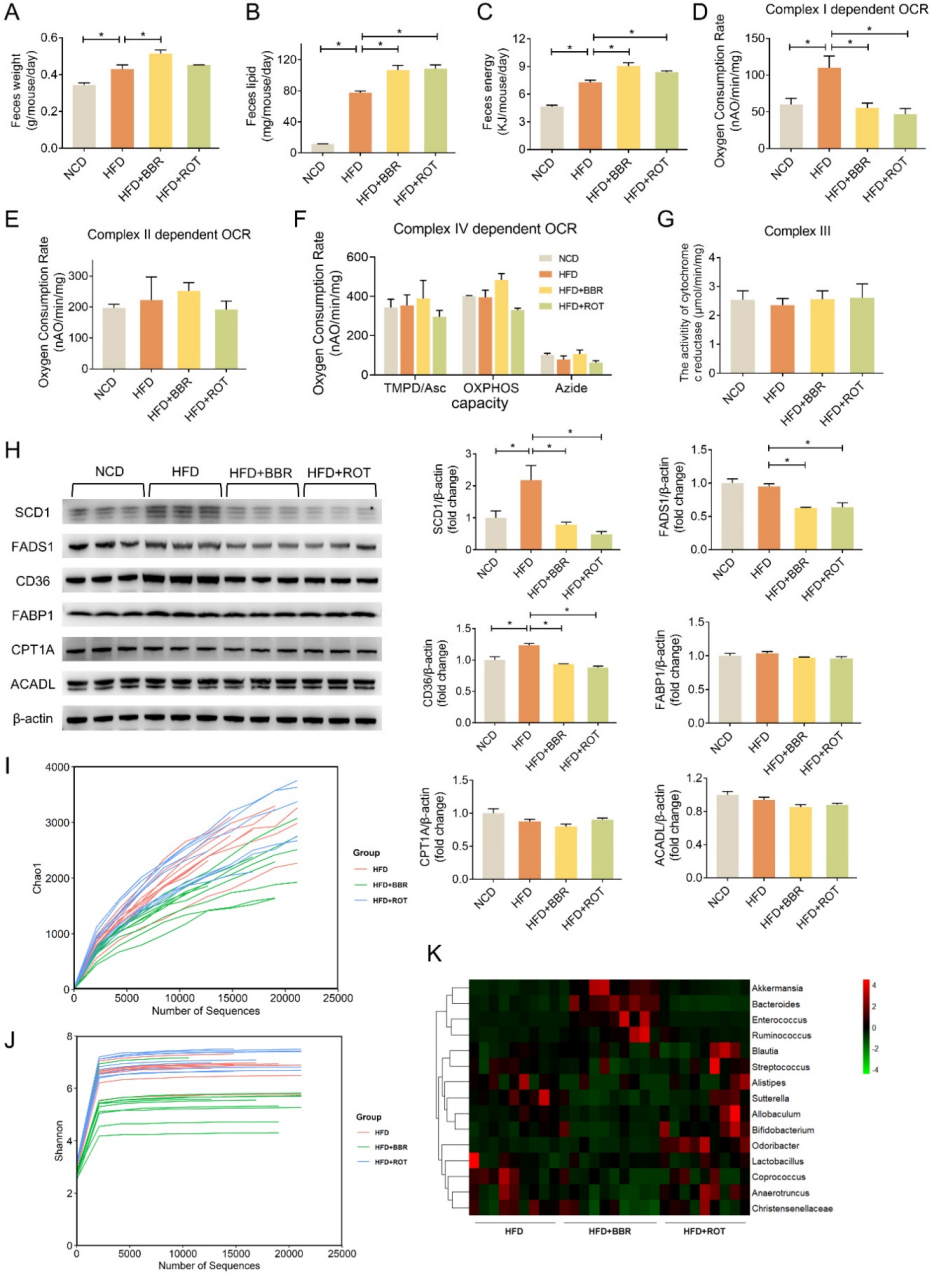
** BBR promoted lipid excretion by inhibiting intestinal complex I.** (A-C) Daily fecal amount (A), lipid excretion (B) and fecal energy (C) of each group in seven consecutive days (*n* = 3). (D-F) The gut mitochondria were extracted, and complex I dependent OCR (D), complex II dependent OCR (E) and complex IV dependent OCR (F) were measured (*n* = 3). (G) The activity of cytochrome c reductase (complex III) of intestine tissues of NFD, HFD, HFD+BBR and HFD+ROT fed mice. (H) The protein levels of representative key-enzymes in lipid pathways of intestinal tissues, such as SCD1 and FADS1 for lipogenesis, FABP1 and CD36 for fatty acid uptake, as well as CPT1A and ACADL for fatty acid oxidation. Relative signal strength was quantified for each band (*n* = 3). (I-K) Chao1 index (I), Shannon index of microbiota (J) and heat map (K) of sequencing 16S V5-V6 variable region of microbiota in each group (*n* = 9-10). Data were expressed as means ± SEM. *P < 0.05.

Collecting daily feces of each group for 2 weeks continuously, we found that the feces weight of HFD mice was 25.8% more than that of NCD. The feces weight as well as fecal lipid and energy excretion of BBR-fed mice exhibited an evident increase compared with obese mice (Figure 7A-C). What's more, HFD dramatically stimulated the intestinal mitochondrial complex I dependent OCR whereas BBR reduced the complex I dependent OCR by 49.4% (Figure 7D). Neither HFD nor BBR had a remarkable impact on the OXPHOS of complex II and IV (Figure 7E and F). In addition, no significant differences were observed among NCD, HFD, HFD+BBR and HFD+ROT groups in the activity of cytochrome c reductase (complex III) in intestinal tissues (Figure 7G).

The effects of BBR on the key enzymes of intestinal lipid pathways were determined by Western blot. The results revealed that HFD markedly stimulated the expression of key enzymes in lipid synthesis (SCD1) and fatty acid uptake (CD36), which could be reversed by BBR and ROT. Meanwhile, HFD could not induce the protein level of FADS1 whereas BBR and ROT reduced its expression compared with HFD. Additionally, the protein expressions of other key enzymes of lipid metabolism, e.g. FABP1, CPT1A and ACADL were found no significant difference among the four groups (Figure 7H). These results indicated that BBR possibly increased the intestinal lipid excretion by inhibiting the protein levels of SCD1 in lipogenesis and CD36 in fatty acid uptake with no influences on the key enzymes of fatty acid oxidation.

To analyze the effects of BBR on gut bacteria, chao1 index and shannon index were used to represent community richness and community diversity, respectively. The data revealed that BBR decreased the richness and diversity of gut microbiome significantly (Figure 7I and J). Among 15 bacteria associated with short-chain fatty acids (SCFA) production, BBR increased the abundance of 4 bacteria (*Akkermansia, Bacteroides, Enterococcus* and *Ruminococcus*) and decreased the abundance of 6 bacteria (*Allobaculum, Anaerotruncus, Bifidobacterium, Christensenellaceae, Coprococcus* and *Sutterella,*) (Figure 7K). In contrast, little effect of ROT on gut microbiome was observed.

**Figure 8:**
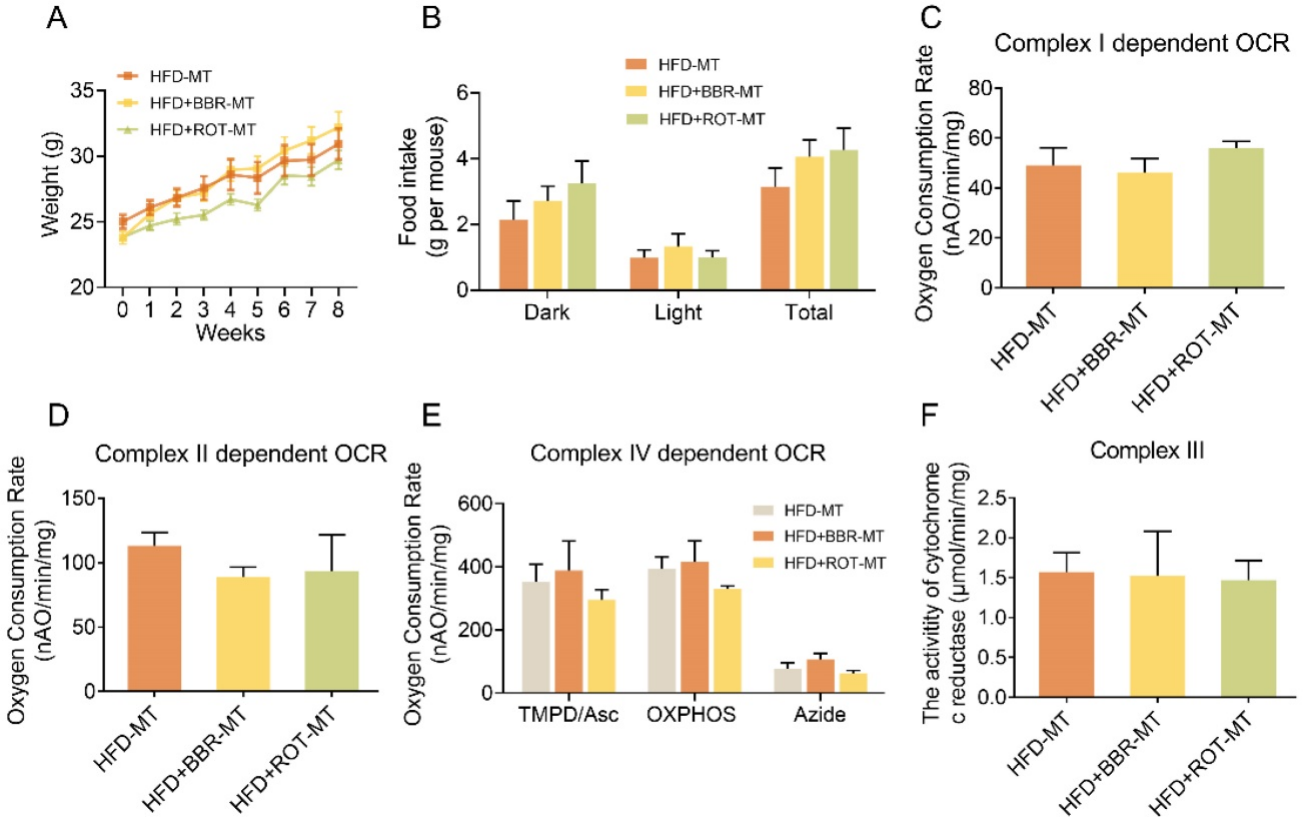
** BBR exerted lipid-lowing effects regardless of the changes in gut microbiome.** (A and B) Body weight (A) and food intake (B) of the recipients after FMT of HFD, HFD+BBR and HFD+ROT (*n* = 3). (C-E) Complex I dependent OCR (C), complex II dependent OCR (D) and complex IV dependent OCR (E) of gut mitochondria in the recipients after the FMT (*n* = 3). (F) The activity of cytochrome c reductase (complex III) of intestinal tissues of the recipients after FMT of HFD, HFD+BBR and HFD+ROT (*n* = 3). Data were expressed as means ± SEM. *P < 0.05.

To clarify the relationship between gut microbiome and the regulatory effects of BBR on metabolism, the feces of HFD, HFD+BBR and HFD+ROT groups were transplanted into C57 mice fed with HFD, respectively. No significant differences were found in body weight, food intake, blood glucose and lipids among the mice after different FMT (Figure 8A, B and Table S2), indicating that FMT of BBR and ROT did not improve the metabolism of obese mice. Furthermore, intestinal mitochondrial OCR and the activity of cytochrome c reductase were similar in all FMT groups (Figure 8C-F), which suggested that the inhibition of mitochondrial complex I was necessary for the amelioration of lipid metabolism disorders by BBR.

## Discussion

This study showed that BBR inhibited mitochondrial complex I-dependent OXPHOS function *in vivo*. Previously, whether oral administration of BBR was able to inhibit liver mitochondrial complex I remained questionable due to its low efficiency of gastrointestinal absorption. This study provided evidence that oral BBR totally reversed the hyperfunction of liver complex I induced by HFD. In primary hepatocytes, both BBR and ROT downregulated the increased ATP content stimulated by OA to normal levels. Above all, both *in vivo* and *in vitro*, BBR completely normalized the energy balance under excessive nutrition.

In addition to mitochondrial function, we found that BBR reduced mtDNA amount and the activity of CS* in vivo*, a rate-limiting enzyme of the tricarboxylic acid cycle, and promoted mitochondrial fusion. Furthermore, BBR ameliorated the mitochondrial swelling induced by HFD. Mitochondrion is a dynamically changing organelle that undergoes fusion and fission under external pressure and metabolism changes, integrating mtDNA to accommodate varying energy requirements 38, 39. Generally, stimuli such as starvation and increased glycolysis could promote mitochondrial fusion to synthesize ATP more efficiently 40, 41. ROT was reported to reduce mtDNA copies of PC12 cells in a dose-dependent manner 42 whereas a decrease of mtDNA copies was also observed in BBR treated K1735-M2 mouse melanoma cells 43. Furthermore, Arnold et al. 44 revealed that high concentration ROT increased the mitochondrial fusion of rat primary cortical neuronal cells. Mitochondria were known to play a decisive role in apoptosis while mitochondrial fusion can increase tolerance to stress and avoid apoptosis or necrosis 45. Notably, effects of BBR on cell death vary depending on cell types and conditions. BBR was reported to promote apoptosis in tumor cells such as non-small-cell lung cancer cells, ovarian cancer cells and human malignant pleural mesothelioma NCI-H2452 cells 46-48. In contrast, BBR significantly reduced the lipoapoptosis on β-cell induced by palmitate and autophagy on H9c2 myocytes caused by hypoxia 49-51. LPS-induced cell death in U251 cells and t-BHP-induced apoptosis in PC12 cells were both attenuated by BBR in a dose-dependent manner 52, 53. That suggested BBR could suppress cytotoxicity induced by different harmful factors. Our study revealed that BBR stimulated mitochondrial fusion, which may contribute to the cytoprotective action of BBR.

This study found that BBR inhibited most key steps of hepatic lipid metabolism, including fatty acid uptake, β-oxidation and lipid synthesis. In addition, BBR could also suppress the key enzymes of intestinal fatty acid uptake and lipid synthesis, which were induced by HFD administration. The finding was contradictory to some previous reports, in which BBR promoted fatty acid oxidation in HFD and db/db mice 24, 25. Different routes of administration and detection methods may be the possible reasons for the discrepancy. Those reports suggested that BBR enhanced fatty acid oxidation in the liver of obese animals which were administered intraperitoneally by the detection of gene expression at mRNA level. However, mRNA levels cannot represent the protein levels, which are mainly resulted from a post-transcriptional regulation 54. In our study, instead of intraperitoneal administration, BBR was mixed into the diet, which was much closer to clinical practice, and the β-oxidation dependent OCR was directly determined in isolated liver mitochondria. The results demonstrated that BBR reduced fatty acid oxidation *in vivo*. Our previous study showed that ROT reduced the ratio of NAD^+^/NADH via complex I inhibition 22. NADH is a product of not only glucose metabolism, but also fatty acid oxidation. Thus, a decrease in the ratio of NAD^+^/NADH might result in the slowdown of fatty acid oxidation and glucose oxidation. In addition, since ATP was indispensable to synthesize lipid, inhibition of ATP production by BBR markedly reduced lipid synthesis. Reduced fatty acid oxidation and triglyceride synthesis further limited fatty acid uptake. Consequently, the functional inhibition of complex I blocked the whole lipid metabolism. In contrast to current understanding, this study suggested the suppression of whole lipid metabolism pathways was able to alleviate hepatic steatosis and dyslipidemia.

No research has discussed the association between BBR and intestinal complex I inhibition in lipid metabolism. In our study, the increased fecal lipids of BBR and ROT groups implied less lipid absorption in the intestine, which may be due to a notable decrease in OCR of the intestinal mitochondrial complex I and protein level of CD36. The above results provide evidence that the intestine is another crucial target organ for lipid metabolism in addition to liver.

No cause-effect relationship was found between the change of gut microbiome and the improvement of lipid metabolism by BBR treatment, which was another important finding of this study. The lipid-lowering effects of BBR and ROT were very similar whereas the effects on gut microbiome were quite different. ROT had little influence on the diversity of gut microbiome, and showed the opposite trend to some bacteria closely related to metabolism compared with BBR. Most of the bacteria in intestinal tract were obligate anaerobic, lacking complete respiratory enzymes, which were the pharmaceutic target of ROT. That may explain why ROT showed little effect on gut microbiome. Thus, complex I inhibition, the common mechanism of BBR and ROT, rather than alteration of gut microbiome, was the real reason of the agents' lipid-lowering effects. Our FMT results further confirmed the hypothesis. FMT of BBR and ROT did not transfer the metabolic improvement to the recipients because FMT could not alter the recipients' mitochondrial function. Our results demonstrated that the action of BBR was not resulted from the alteration of gut microbiome.

When it comes to the safety of complex I inhibition, BBR is an over-the-counter drug for the treatment of bacterial diarrhea in China. In USA and other countries, BBR is usually sold as a dietary supplement. In addition, several clinical trials including ours proved that the most common adverse events of BBR were gastrointestinal discomfort 7, 55, 56 and its severe adverse effects have never been reported. ROT has not been used in human studies whereas our data showed that it did not exert a harmful effect on the mice as evidenced by normal food intake and hepatorenal function, which was consistent with BBR, indicating the moderate complex I inhibition might be in good safety for clinical application.

## Conclusion

**Figure 9:**
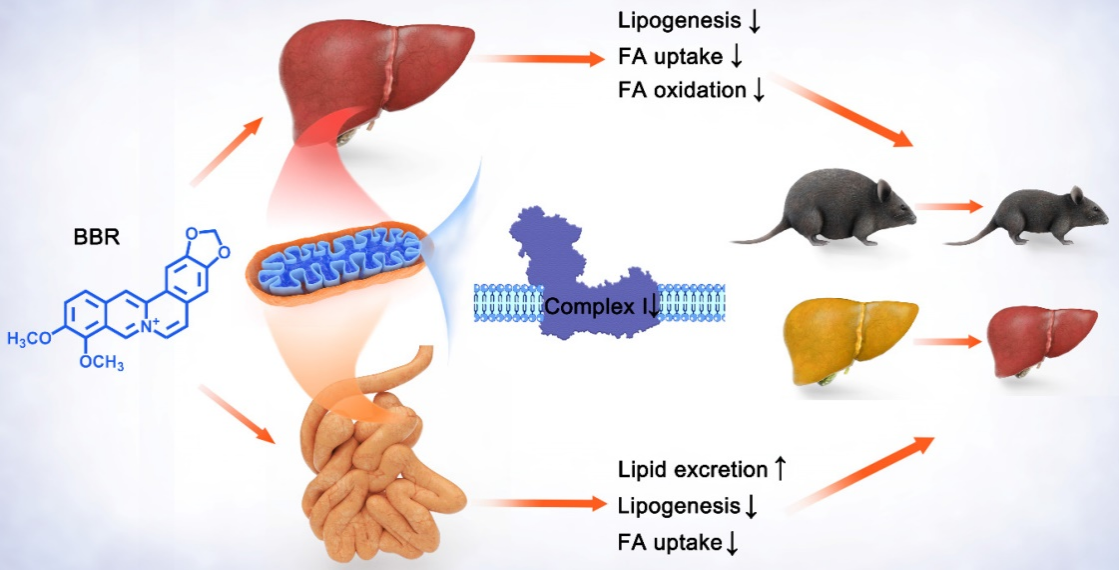
** Schematic diagram depicting the role of mitochondrial complex I inhibition in the lipid-lowing effects of BBR.** BBR downregulates the hyperfunction of mitochondrial complex I induced by energy excess. Its inhibitory effect on gut leads to increased lipid excretion with reduced fatty acid uptake and lipogenesis, while the decrease of lipogenesis, fatty acid uptake and oxidation is shown in liver. These alterations by BBR ameliorate obesity, fatty liver and insulin resistance.

To sum up, these findings indicated that BBR and ROT inhibited the mitochondrial electron transport chain complex I of gut and liver, and led to the suppression of lipid metabolism including intestinal fatty acid uptake and lipogenesis, as well as hepatic fatty acid uptake, β-oxidation and lipid synthesis. Consequently, obesity, insulin resistance and hepatic steatosis were reversed by BBR and ROT in the dietary obese mice (Figure 9). Considering the similar efficacy of BBR and ROT on metabolism, we suggest mitochondrial complex I as a promising drug target for obesity.

## Supplementary Material

Supplementary tables.

## References

[1] Saltiel AR (2016). New therapeutic approaches for the treatment of obesity. Sci Transl Med.

[2] Swinburn B, Dietz W, Kleinert S (2015). A Lancet Commission on obesity. Lancet.

[3] Khera R, Murad MH, Chandar AK (2016). Association of Pharmacological Treatments for Obesity With Weight Loss and Adverse Events: A Systematic Review and Meta-analysis. JAMA.

[4] Rodgers RJ, Tschöp MH, Wilding JP (2012). Anti-obesity drugs: past, present and future. Dis Model Mech.

[5] Heymsfield SB, Wadden TA (2017). Mechanisms, Pathophysiology, and Management of Obesity. N Engl J Med.

[6] Wang Y, Campbell T, Perry B (2011). Hypoglycemic and insulin-sensitizing effects of berberine in high-fat diet- and streptozotocin-induced diabetic rats. Metabolism.

[7] Yin J, Xing H, Ye J (2008). Efficacy of berberine in patients with type 2 diabetes mellitus. Metabolism.

[8] Yin J, Hu R, Chen M (2002). Effects of berberine on glucose metabolism *in vitro*. Metabolism.

[9] Chen Y, Li Y, Wang Y (2009). Berberine improves free-fatty-acid-induced insulin resistance in L6 myotubes through inhibiting peroxisome proliferator-activated receptor gamma and fatty acid transferase expressions. Metabolism.

[10] Sun Y, Yu J, Liu X (2018). Oncosis-like cell death is induced by berberine through ERK1/2-mediated impairment of mitochondrial aerobic respiration in gliomas. Biomed Pharmacother.

[11] Kong WJ, Wei J, Zuo ZY (2008). Combination of simvastatin with berberine improves the lipid-lowering efficacy. Metabolism.

[12] Wang Y, Yi X, Ghanam K (2014). Berberine decreases cholesterol levels in rats through multiple mechanisms, including inhibition of cholesterol absorption. Metabolism.

[13] Buchner DA, Yazbek SN, Solinas P (2011). Increased mitochondrial oxidative phosphorylation in the liver is associated with obesity and insulin resistance. Obesity (Silver Spring).

[14] Guo Y, Darshi M, Ma Y (2013). Quantitative proteomic and functional analysis of liver mitochondria from high fat diet (HFD) diabetic mice. Mol Cell Proteomics.

[15] Alimujiang M, Yu X, Yu M (2020). Enhanced liver but not muscle OXPHOS in diabetes and reduced glucose output by complex I inhibition. J Cell Mol Med.

[16] Xia X, Yan J, Shen Y (2011). Berberine improves glucose metabolism in diabetic rats by inhibition of hepatic gluconeogenesis. PLoS One.

[17] Xu M, Xiao Y, Yin J (2014). Berberine promotes glucose consumption independently of AMP-activated protein kinase activation. PLoS One.

[18] Yin J, Gao Z, Liu D (2008). Berberine improves glucose metabolism through induction of glycolysis. Am J Physiol Endocrinol Metab.

[19] Sun Y, Yuan X, Zhang F (2017). Berberine ameliorates fatty acid-induced oxidative stress in human hepatoma cells. Sci Rep.

[20] Yan XJ, Yu X, Wang XP (2017). Mitochondria play an important role in the cell proliferation suppressing activity of berberine. Sci Rep.

[21] Turner N, Li JY, Gosby A (2008). Berberine and its more biologically available derivative, dihydroberberine, inhibit mitochondrial respiratory complex I: a mechanism for the action of berberine to activate AMP-activated protein kinase and improve insulin action. Diabetes.

[22] Hou WL, Yin J, Alimujiang M (2018). Inhibition of mitochondrial complex I improves glucose metabolism independently of AMPK activation. J Cell Mol Med.

[23] Knottnerus SJG, Bleeker JC, Wüst RCI (2018). Disorders of mitochondrial long-chain fatty acid oxidation and the carnitine shuttle. Rev Endocr Metab Disord.

[24] Brusq JM, Ancellin N, Grondin P (2006). Inhibition of lipid synthesis through activation of AMP kinase: an additional mechanism for the hypolipidemic effects of berberine. J Lipid Res.

[25] Kim WS, Lee YS, Cha SH (2009). Berberine improves lipid dysregulation in obesity by controlling central and peripheral AMPK activity. Am J Physiol Endocrinol Metab.

[26] Yu Y, Raka F, Adeli K (2019). The Role of the Gut Microbiota in Lipid and Lipoprotein Metabolism. J Clin Med.

[27] Sun H, Wang N, Cang Z (2016). Modulation of Microbiota-Gut-Brain Axis by Berberine Resulting in Improved Metabolic Status in High-Fat Diet-Fed Rats. Obes Facts.

[28] Zhu L, Zhang D, Zhu H (2018). Berberine treatment increases Akkermansia in the gut and improves high-fat diet-induced atherosclerosis in Apoe-/- mice. Atherosclerosis.

[29] Agustí A, García-Pardo MP, López-Almela I (2018). Interplay Between the Gut-Brain Axis, Obesity and Cognitive Function. Front Neurosci.

[30] Luo H, Jiang M, Lian G (2018). AIDA Selectively Mediates Downregulation of Fat Synthesis Enzymes by ERAD to Retard Intestinal Fat Absorption and Prevent Obesity. Cell Metab.

[31] Frezza C, Cipolat S, Scorrano L (2007). Organelle isolation: functional mitochondria from mouse liver, muscle and cultured fibroblasts. Nat Protoc.

[32] Nanadikar MS, Vergel Leon AM, Borowik S (2019). O_2_ affects mitochondrial functionality *ex vivo*. Redox Biol.

[33] Chen Q, Hoppel CL, Lesnefsky EJ (2006). Blockade of electron transport before cardiac ischemia with the reversible inhibitor amobarbital protects rat heart mitochondria. J Pharmacol Exp Ther.

[34] Berry MN, Friend DS (1969). High-yield preparation of isolated rat liver parenchymal cells: a biochemical and fine structural study. J Cell Biol.

[35] Guo T, Woo SL, Guo X (2016). Berberine Ameliorates Hepatic Steatosis and Suppresses Liver and Adipose Tissue Inflammation in Mice with Diet-induced Obesity. Sci Rep.

[36] Thomas SS, Plenkiewicz J, Ison ER (1995). Influence of monosaccharide derivatives on liver cell glycosaminoglycan synthesis: 3-deoxy-D-xylo-hexose (3-deoxy-D-galactose) and methyl (methyl 4-chloro-4-deoxy-beta-D-galactopyranosid) uronate. Biochim Biophys Acta.

[37] Harach T, Pols TW, Nomura M (2012). TGR5 potentiates GLP-1 secretion in response to anionic exchange resins. Sci Rep.

[38] Hyde BB, Twig G, Shirihai OS (2010). Organellar vs cellular control of mitochondrial dynamics. Semin Cell Dev Biol.

[39] Knott AB, Bossy-Wetzel E (2008). Impairing the mitochondrial fission and fusion balance: a new mechanism of neurodegeneration. Ann N Y Acad Sci.

[40] Meyer JN, Leuthner TC, Luz AL (2017). Mitochondrial fusion, fission, and mitochondrial toxicity. Toxicology.

[41] Silva Ramos E, Larsson NG, Mourier A (2016). Bioenergetic roles of mitochondrial fusion. Biochim Biophys Acta.

[42] Peng K, Yang L, Wang J (2017). The Interaction of Mitochondrial Biogenesis and Fission/Fusion Mediated by PGC-1α Regulates Rotenone-Induced Dopaminergic Neurotoxicity. Mol Neurobiol.

[43] Pereira GC, Branco AF, Matos JA (2007). Mitochondrially targeted effects of berberine [Natural Yellow 18, 5,6-dihydro-9,10-dimethoxybenzo(g)-1,3-benzodioxolo(5,6-a) quinolizinium] on K1735-M2 mouse melanoma cells: comparison with direct effects on isolated mitochondrial fractions. J Pharmacol Exp Ther.

[44] Arnold B, Cassady SJ, VanLaar VS (2011). Integrating multiple aspects of mitochondrial dynamics in neurons: age-related differences and dynamic changes in a chronic rotenone model. Neurobiol Dis.

[45] Westermann B (2010). Mitochondrial fusion and fission in cell life and death. Nat Rev Mol Cell Biol.

[46] Chen QQ, Shi JM, Ding Z (2019). Berberine induces apoptosis in non-small-cell lung cancer cells by upregulating miR-19a targeting tissue factor. Cancer Manag Res.

[47] Liu L, Fan J, Ai G (2019). Berberine in combination with cisplatin induces necroptosis and apoptosis in ovarian cancer cells. Biol Res.

[48] Yao Z, Wan Y, Li B (2018). Berberine induces mitochondrial-mediated apoptosis and protective autophagy in human malignant pleural mesothelioma NCI-H2452 cells. Oncol Rep.

[49] Gao N, Zhao TY, Li XJ (2011). The protective effect of berberine on β-cell lipoapoptosis. J Endocrinol Invest.

[50] Li J, Du H, Zhang M (2019). Amorphous solid dispersion of Berberine mitigates apoptosis via iPLA2β/Cardiolipin/Opa1 pathway in db/db mice and in Palmitate-treated MIN6 β-cells. Int J Biol Sci.

[51] Jia Z, Lin L, Huang S (2017). Inhibition of autophagy by berberine enhances the survival of H9C2 myocytes following hypoxia. Mol Med Rep.

[52] Yu X, Wang S, Wang J (2020). Berberine Induces CYP2J2 Expression in Human U251 Glioma Cells via Regulation of Peroxisome Proliferator-Activated Receptor Alpha. Pharmacology.

[53] Li Z, Jiang T, Lu Q (2020). Berberine attenuated the cytotoxicity induced by t-BHP via inhibiting oxidative stress and mitochondria dysfunction in PC-12 cells. Cell Mol Neurobiol.

[54] Tajima K, Ikeda K, Chang HY (2019). Mitochondrial lipoylation integrates age-associated decline in brown fat thermogenesis. Nat Metab.

[55] Yan HM, Xia MF, Wang Y (2015). Efficacy of Berberine in Patients with Non-Alcoholic Fatty Liver Disease. PLoS One.

[56] Spigoni V, Aldigeri R, Antonini M (2017). Effects of a New Nutraceutical Formulation (Berberine, Red Yeast Rice and Chitosan) on Non-HDL Cholesterol Levels in Individuals with Dyslipidemia: Results from a Randomized, Double Blind, Placebo-Controlled Study. Int J Mol Sci.

