# Supplementary material for: Berberine alleviates lipid metabolism disorders via inhibition of mitochondrial complex I in gut and liver
Source: Int J Biol Sci. 2021 Apr 12;17(7):1693–707. doi: 10.7150/ijbs.54604 (PMC8120465; doi:10.7150/ijbs.54604)
Supplement: Supplementary file 1 — Supplementary tables. [file ijbsv17p1693s1.pdf]

Table S1. Effects of BBR and ROT on serum biochemical indexes of diet-induced mice

	NCD	HFD	HFD+BBR	HFD+ROT
ALT (U/L)	40.17±2.12	90.33±25.51*	51.17±7.78	49.17±15.08
Creatinine (μmol/L)	8.83±0.87	7.20±1.07	4.20±0.97	5.17±1.40
HDL-C (mmol/L)	1.25±0.12	1.54±0.03	1.53±0.05	1.44±0.06
LDL-C (mmol/L)	0.23±0.02	0.28±0.04	0.25±0.03	0.25±0.04
Uric acid (mmol/L)	85.67±15.71	108.70±16.79	125.00±23.00	118.70±21.51

Data were expressed as means ± SEM (n = 6). **P* < 0.05 vs. NCD.

Table S2. Effects of fecal transplantation on serum biochemical indexes of mice

	HFD-MT	BBR-MT	ROT-MT
ALT (U/L)	35.83±11.21	27.33±2.39	28.17±1.66
Cholesterol (mmol/L)	3.12±0.39	3.23±0.32	3.30±0.28
Creatinine (μmol/L)	9.33±2.19	7.50±0.67	7.50±0.76
Glucose (mg/dL)	277.20±25.52	274.86±11.27	268.92±19.49
HDL-C (mmol/L)	1.97±0.07	1.98±0.07	2.02±0.13
LDL-C (mmol/L)	0.21±0.03	0.22±0.02	0.22±0.01
Triglyceride (mmol/L)	0.63±0.06	0.58±0.06	0.50±0.04
Urea (mmol/L)	57.17±2.41	59.83±12.45	46.00±11.43

Data were expressed as means ± SEM (n = 6).
